# Immune complexes-mediated activation of neutrophils in systemic lupus erythematosus is dependent on RNA recognition by toll-like receptor 8

**DOI:** 10.3389/fimmu.2024.1515469

**Published:** 2024-12-24

**Authors:** Ting Wang, Runa Kuley, Payton Hermanson, Peirou Chu, Christopher Pohlmeyer, Jayamary Divya Ravichandar, David Lopez, Gundula Min-Oo, Natasha Crellin, Ching Shang, Christian Lood

**Affiliations:** ^1^ Division of Rheumatology, University of Washington, Seattle, WA, United States; ^2^ Centre for Life Sciences, Mahindra University, Hyderabad, India; ^3^ Inflammation Research, Gilead Sciences, Inc., Foster City, CA, United States; ^4^ Inflammation Department, Amgen Inc., Thousand Oaks, CA, United States

**Keywords:** immune complexes, neutrophils, systemic lupus erythematosus, toll-like receptor 8, RNA recognition

## Abstract

**Introduction:**

Neutrophil activation is important in systemic lupus erythematosus (SLE). We previously demonstrated that ribonucleoprotein (RNP) immune complexes (ICs) promoted neutrophil activation in a TLR7/8-dependent manner. However, it remains unclear if this mechanism occurs in patients. Here, we investigated the role of RNA recognition by evaluating TLR7/8 in plasma-mediated neutrophil activation in SLE.

**Methods:**

Plasma levels of neutrophil activation markers and ICs were measured by ELISA and flow cytometry in SLE patients (n=151) and healthy controls (HCs, n=31). Neutrophils were incubated with plasma and assessed for CD66b and CD11b up-regulation by flow cytometry in the presence of select inhibitors to define the mechanisms of neutrophil activation by SLE plasma.

**Results:**

SLE plasma induced higher levels of CD66b (p=0.0002) and CD11b (p=0.01) expression than plasma from HCs. Blocking FcγRIIA, targeting RNA sensing by adding RNase, or blocking TLR7/8, TLR8 only, or IRAK4, decreased plasma-mediated neutrophil activation (p<0.05). Consistent with the ability of selective TLR8 inhibitor to block plasma-mediated neutrophil activation, TLR8 agonists, but not TLR7 agonists induced robust neutrophil activation. Further, neutrophil mRNA expression of TLR8 was higher than TLR7. Finally, patients with plasma samples inducing neutrophil activation in RNA-dependent manner had increased levels of interferon alpha, IP-10 (p<0.05), ICs (p<0.05), and reduced complement C3 levels (p<0.01), indicative of IC-driven disease.

**Conclusion:**

The data support IC-driven RNA-sensing by TLR8 in neutrophils is a key mechanism of neutrophil activation in SLE. Patients with elevated neutrophil activation and presence of RNA-containing ICs, may benefit from TLR8 inhibition and other strategies targeting RNA removal.

## Introduction

SLE is a heterogenous, multiple organ-involved, potentially life-threatening autoimmune disease characterized by the production of pathogenic anti-nuclear autoantibodies (ANAs) and the formation of immune complexes (ICs). The pathogenesis of SLE is complex, involving environmental factors, genetic changes, and loss of immune tolerance (1). So far, the exact etiology of SLE is not fully understood.

Neutrophils, the most abundant immune cells in circulation, play a crucial role in host defense through phagocytosis, production of reactive oxygen species (ROS), and the formation of neutrophil extracellular traps (NETs). While these functions are beneficial in combating pathogens, excessive neutrophil activation and NET formation have been linked to inflammation and autoimmunity (2–6), including in SLE (7). Once released, NETs are a prominent source of autoantigens (8), B cell activating factor, BAFF (7), and proinflammatory cytokines, including interferons (9), key cytokines in SLE pathogenesis. Levels of NETs are increased in SLE and associated with disease severity, including nephritis and cardiovascular disease, and can predict upcoming disease flares (10).

Neutrophil activation and NET formation can be triggered by microbial components, as well as by sterile stimuli including cytokines, ICs, and autoantibodies (11); *in vitro*, they can also be induced by phorbol myristate acetate (PMA) or calcium ionophore (12). We previously demonstrated that ribonucleoprotein (RNP) ICs promoted neutrophil activation and NET formation in a TLR7/8-dependent manner (13). Still, whether this process occurs in patients has not been clearly defined.

In this study, we investigated the role of TLR7/8 in SLE plasma-mediated neutrophil activation. In brief, we found that SLE plasma induced neutrophil activation in an FcγRIIA- and TLR8-dependent manner. Thus, in patients with increased neutrophil activation, targeting the RNA component of ICs, such as by TLR8 inhibition, should be considered.

## Methods

### Study subjects

SLE patients (n = 151, cohort 1) were recruited from the University of Washington Rheumatology Biorepository. All patients fulfilled the 1982 revised ACR criteria for SLE (14). Disease activity was measured using the SLE Disease Activity Index (SLEDAI) 2K. Additionally, age -matched healthy controls (HC) (n = 31) with no history of autoimmune diseases or current infections were included. **Supplementary Table 1** displays the demographic and clinical characteristics of patients enrolled. The study was approved by the University of Washington review board, and informed consent was obtained from all participants in accordance with the Helsinki Declaration.

### ELISAs

Freshly isolated plasma samples were aliquoted and stored at −80°C until use. Plasma levels of calprotectin were analyzed using a commercial ELISA kit (R&D Systems, Minneapolis, MN, USA). Plasma myeloperoxidase (MPO)-DNA and neutrophil elastase (NE)-DNA complexes levels were quantified with in-house ELISAs, as described previously (4, 5).

### Immune complexes quantification

FcγRIIA internalization or occupation, a bioassay for IC quantification, was analyzed by flow cytometry as described (15). In brief, neutrophils were incubated with plasma samples, and IC was quantified as loss of cell surface FcγRIIA expression as determined by flow cytometry.

### Neutrophil activation and IL-8 production

Neutrophils were isolated from healthy subjects with Polymorphprep (Axis-Shield, Dundee, UK) as described previously (7, 16). Neutrophils were activated with R848 (InvivoGen, San Diego, CA, USA), CL075 (InvivoGen, San Diego, CA, USA), Imiquimod (InvivoGen, San Diego, CA, USA), or plasma (1:50 dilution) from SLE patients or HCs, for 2h, with or without prior addition of a TLR7/8 inhibitor (Example 438 from US 2019/0185469, provided by Gilead Sciences), a TLR7/8/9 inhibitor (Example 50 from WO 2019/238616, provided by Gilead Sciences), an IRAK4 inhibitor (provided by Gilead Sciences, Foster City, CA, USA), CU-CPT9a (TLR8 inhibitor, InvivoGen, San Diego, CA, USA), RNase A (Thermo Fisher Scientific, Waltham, MA, USA), FcγR II inhibitor (IV.3; Caprico Biotechnologies, Norcross, GA, USA) and hydroxychloroquine sulfate (HCQ, Sigma-Aldrich, Louis and Burlington, MA, USA) for 60 min. Activation was assessed by flow cytometer (Beckman Coulter, Brea, CA, USA) by assessing cell surface levels of CD66b (clone G10F5, BioLegend) and CD11b (clone CBRM1/5, BioLegend, San Diego, CA USA). Data were analyzed by FlowJo (Tree Star, Inc., Ashland, OR, USA). For IL-8 production, neutrophils or peripheral blood mononuclear cells (PBMCs) were isolated from healthy donors and incubated with appropriate stimuli with or without prior addition of different inhibitors. After overnight incubation, cell culture supernatants were collected and measured for IL-8 production with ELISA (Biolegend, San Diego, CA USA) following the manufacturer’s instructions.

### Cytokine measurement

Serum levels of IFN-λ1 and IFNα2a were measured with a custom-developed MSD^®^ S-plex^®^ Human multiplex panel at Meso Scale Discovery (Rockville, MD, USA). Serum levels of IP-10 (CXCL10) were measured with the MSD^®^ U-plex^®^ assay (Meso Scale Discovery, Rockville, MD USA) according to the manufacturer’s instructions.

### RT-qPCR

Total RNA was isolated from human neutrophils from healthy donors (n=13) and SLE patients (n=13, cohort 2) using Quick-RNA from Whole Blood (ZymoResearch), followed by reverse transcription into cDNA using N High-Capacity cDNA Prep (Applied Biosystems). Reactions were performed in duplicate on an ABI StepOne Plus instrument using appropriate primers (**Supplementary Table 2**). A 2-stage cycle of 95°C for 15 seconds and 60°C for 1 minute was repeated for 40 cycles followed by a dissociation stage.

### Statistical analysis

Statistical tests were performed using GraphPad Prism 10.0 (GraphPad, San Diego, CA, USA). For sample sets with non-Gaussian distribution, non-parametric tests, Mann‐Whitney U test, and Wilcoxon’s paired test were used when applicable. P values less than 0.05 were considered significant.

## Results

### SLE plasma induces neutrophil activation in a TLR7/8-dependent manner

**Figure 1:**
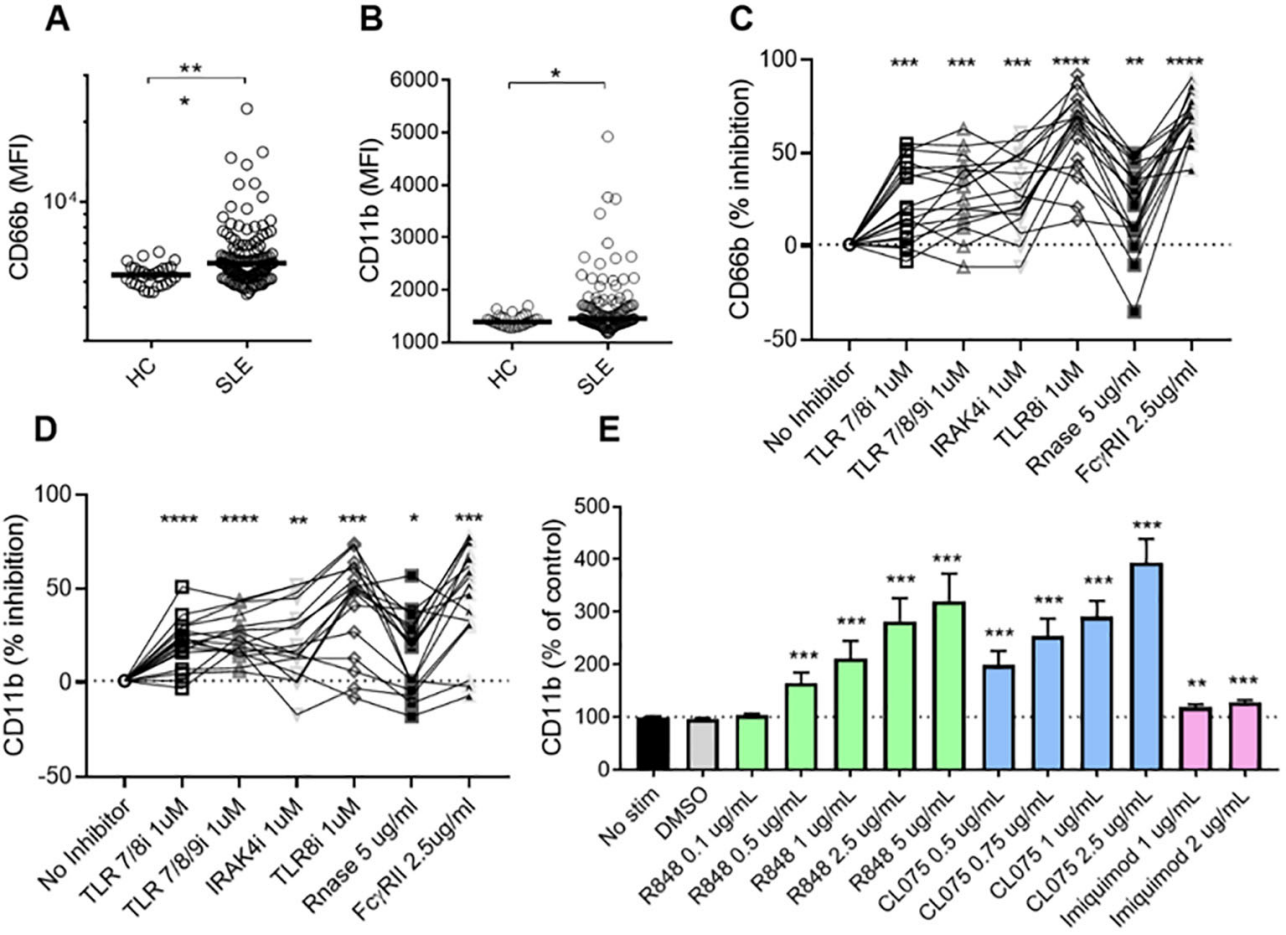
SLE plasma triggers neutrophil activation in a TLR7/8-dependent manner. Neutrophils isolated from healthy donors were incubated with plasma from HCs (n=29) or SLE patients (n=120) for 2 hours, and analyzed for cell surface expression of CD66b **(A)** and CD11b **(B)** by flow cytometry. Results are presented as the mean fluorescence intensity (MFI). Statistical analyses were done using the Mann–Whitney U-test. Each circle represents an individual sample, with the bar representing the group's median. Neutrophils were activated with SLE plasma (n=17) for 2h, with or without prior addition of TLR7/8 inhibitor (TLR7/8i), TLR7/8/9 inhibitor (TLR7/8/9i), IRAK4 inhibitor (IRAK4i), TLR8 inhibitor (TLR8i), RNase A, and Fcγ receptor II inhibitor (IV.3) for 60 min as described in the Methods section. Activation was assessed by flow cytometry by assessing cell surface levels of CD66b **(C)** and CD11b **(D)**. For Figures **(C, D)**, samples (n=17) were selected from the plasma samples inducing the highest levels of CD66b expression, based on data from **(A)**. Results are presented as % inhibition as compared to no inhibitor added. Statistical analyses were done using Wilcoxon’s paired test. Neutrophils were activated with R848 (TLR7/8 agonist), CL075 (TLR7/8 agonist), and Imiquimod (TLR7 agonist) at different doses for 2 hours before assessed by flow cytometry for cell surface levels of CD11b **(E)**. The experiment was repeated 6 times and combined results are shown and compared with no stimuli using a paired Wilcoxon test. For all tests, *p < 0.05, **p < 0.01, ***p < 0.001, and ****p <0.0001.

Plasma levels of calprotectin, MPO-DNA, and NE-DNA complexes were significantly higher in patients with SLE as compared to HCs (**Supplementary Figure 1**), indicating neutrophil hyperactivation in SLE. To identify the factors triggering neutrophil activation in the circulation, we isolated neutrophils from healthy donors and incubated them with plasma from either SLE patients or HCs. Plasma from SLE patients induced higher levels of cell surface CD66b (p=0.0002) and CD11b (p=0.01) expression compared to plasma from HCs (**Figures 1A, B**). These findings suggest that soluble components in SLE circulation can induce *de novo* neutrophil activation. Given that we previously demonstrated that RNP ICs promoted neutrophil activation in a TLR7/8-dependent manner (13), we wanted to determine whether soluble RNA-containing ICs in plasma contribute to neutrophil activation. As expected, targeting ICs by blocking TLR7/8, TLR7/8/9, TLR8 only, or IRAK4, and/or targeting RNA sensing by adding RNase, and by inhibiting FcγRIIA, decreased the plasma-mediated neutrophil activation (all p<0.05, **Figures 1C, D**), implying that IC-mediated neutrophil activation in SLE is dependent on RNA recognition by TLR7/8.

### Neutrophils are activated by TLR8 agonists, but not TLR7

**Figure 2:**
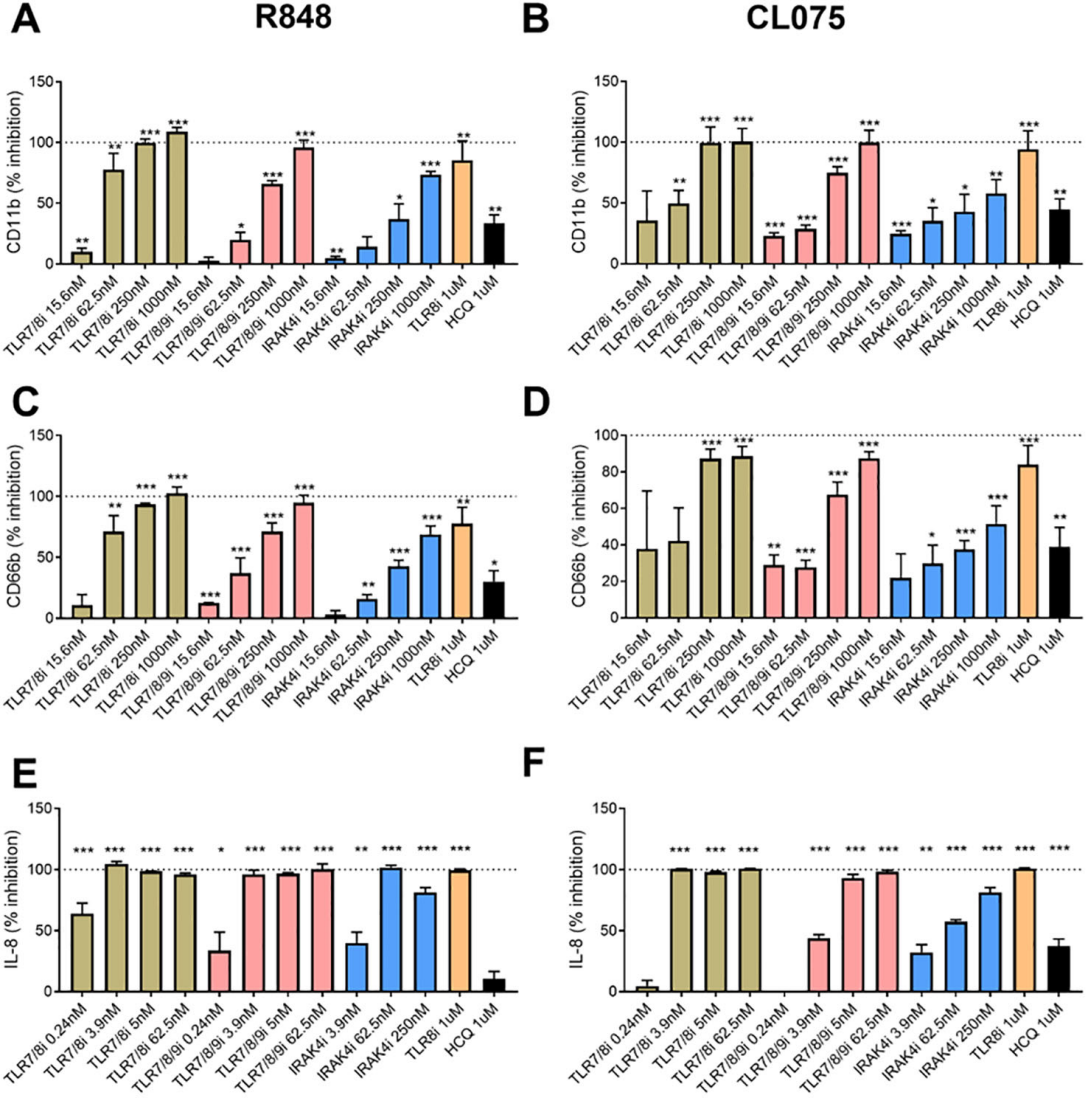
Neutrophil activation with TLR7/8 agonists. Neutrophils were activated with R848 (1 µg/ml, TLR7/8 agonist, **A** and **C**) and CL075 (1 µg/ml, TLR7/8 agonist, **B** and **D**) for 2h **(A-D)** or overnight **(E, F)**, with or without prior addition of TLR7/8 inhibitor (TLR7/8i), TLR7/8/9 inhibitor (TLR7/8/9i), IRAK4 inhibitor (IRAK4i), TLR8 inhibitor (TLR8i), and HCQ for 60 min as described in the Methods section. Neutrophil activation was assessed by flow cytometry by assessing cell surface levels of CD11b **(A, B)** and CD66b **(C, D)**, while IL-8 production were determined by ELISA with collected cell culture supernatants. The experiment was repeated 2 (IL-8) and 4 (CD11b/CD66b) times and combined results are shown and compared with medium control. The results are presented as % inhibition as compared to no inhibitor added. Statistical analyses were done using the Wilcoxon paired test; *p < 0.05, **p < 0.01, and ***p < 0.001.

**Figure 3:**
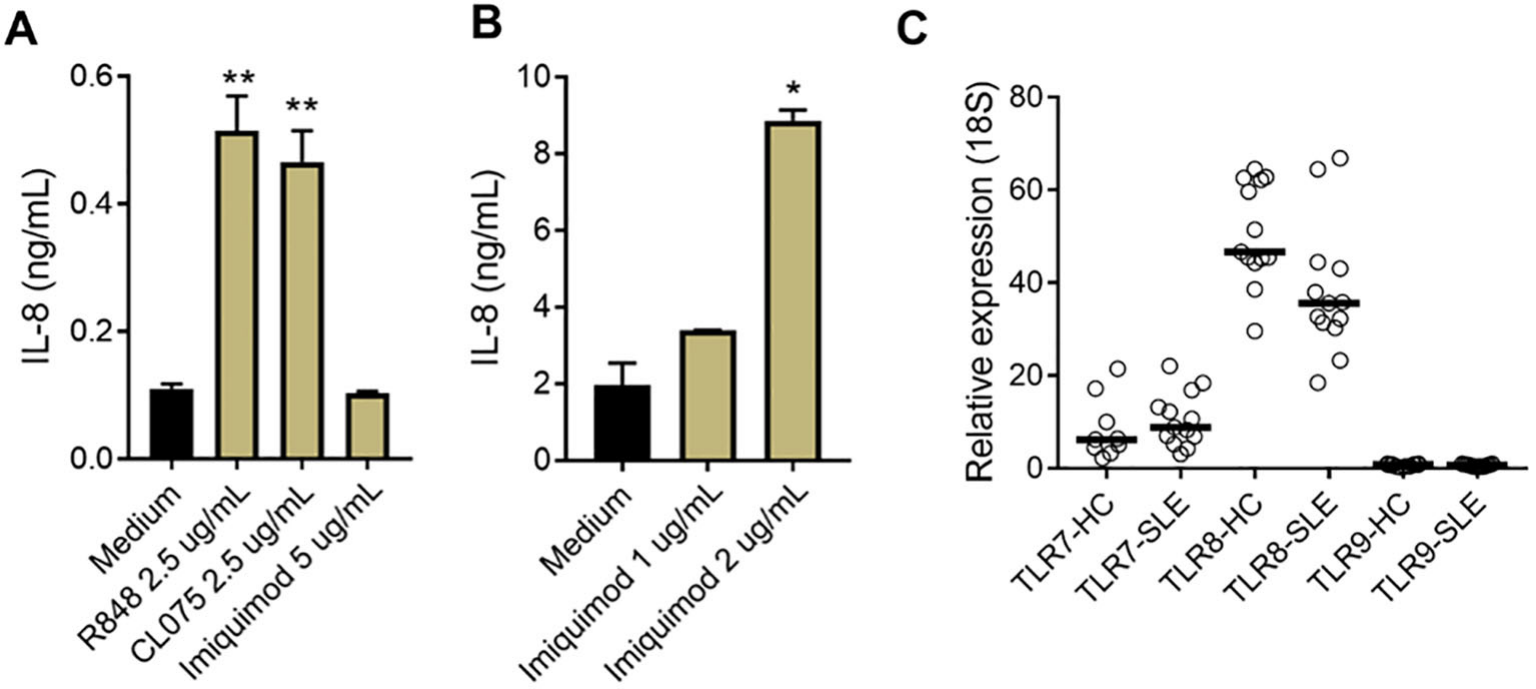
Limited TLR7 response in neutrophils. **(A)** Neutrophils and B) PBMCs were isolated from SLE patients and healthy donors, and incubated with R848, CL075, and Imiquimod, at different concentrations. After overnight incubation, cell culture supernatants were collected and measured for IL-8 production with ELISA. The experiment was repeated at least two times and combined results are shown and compared with medium control using paired Wilcoxon test; *p < 0.05, **p < 0.01. **(C)** TLR7, 8, and 9 mRNA expression in neutrophils from HC (n=13) and SLE patients (n=13) were determined by RT-qPCR.

To further investigate the sensor for RNA-containing ICs in neutrophils, we used dual TLR7/8 agonists (R848 and CL075), and TLR7 agonist (Imiquimod) to stimulate neutrophils. Consistent with the ability of selective TLR8 inhibitors to block plasma-mediated neutrophil activation (**Figures 1C, D**), dual TLR7/8 agonists (R848 and CL075) induced CD11b expression (dose-dependent, **Figure 1E**), while TLR7 agonist (Imiquimod) induced modest to none CD11b expression (**Figure 1E**) when used at similar concentrations as dual TLR7/8 agonists. Similar results were seen for induction of CD66b (data not shown). Moreover, R848 and CL075 induced-neutrophil activation (**Figures 2A–D**), and IL-8 production (**Figures 2E, F**) was blocked by TLR7/8, TLR7/8/9, TLR8 only, or IRAK4 inhibitors in a dose-dependent manner. To note, while dual TLR7/8 agonists (R848 and CL075) significantly induced IL-8 production, TLR7 agonist (Imiquimod) only failed to induce IL-8 production in neutrophils (**Figure 3A**). However, Imiquimod induced robust IL-8 production in PBMC in a dose-dependent manner (**Figure 3B**), demonstrating its capacity to activate TLR7 in PBMCs. Thus, we wondered whether the RNA sensor expressed in human neutrophils was predominantly TLR8 but not TLR7. As expected, neutrophil mRNA expression of TLR8 was higher than that of TLR7 (**Figure 3C**) in neutrophils from both HC and SLE, skewing RNA-sensing to TLR8 in neutrophils. The DNA sensing receptor TLR9 was expressed at very low or undetectable levels in human neutrophils.

### Presence of RNA components is associated with IC-mediated inflammation

**Figure 4:**
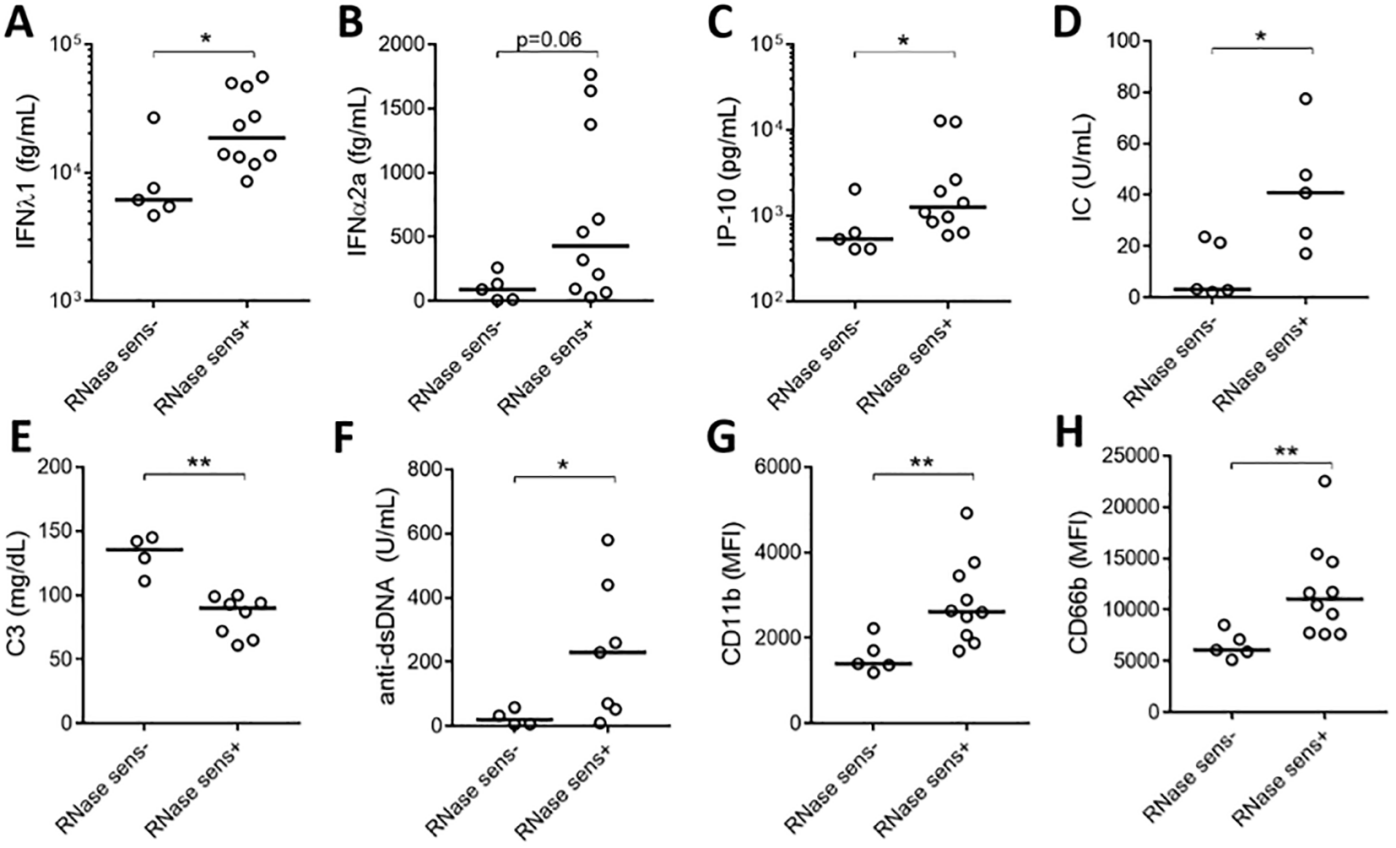
Plasma RNase sensitivity is associated with inflammation. Serum levels of IFN-λ1 **(A)**, IFNα2a **(B)**, and IP-10 **(C)** in RNase-sensitive (RNase sens+, n=10) patients and RNase-insensitive (RNase sens-, n=5) patients. RNase-sensitive patients were defined as those with the presence of RNase sensitive components (including RNA-containing ICs) in their plasma. Circulating IC levels **(D)** in RNase sens+ (n=5) and RNase sens- (n=5) groups as determined by flow cytometry. Complement C3 **(E)** and anti-dsDNA antibody **(F)** levels were obtained from clinical records and compared between RNase sens+ (n=8) and RNase sens- (n=4) groups. Neutrophils were stimulated with RNase sens+ (n=10) and RNase sens- (n=5) patients plasma for 2h and assessed for CD11b **(G)** and CD66b **(H)** expression. Samples or patients (n=17) were selected from the plasma samples inducing the highest levels of CD66b expression, based on data from **Figure 1A**. Statistical analyses were done using the Mann–Whitney U-test; *p < 0.05 and **p < 0.01. Each circle represents an individual sample, with the bar representing the median of the group.

To assess the clinical significance of having soluble RNA ICs, SLE patients were stratified based on their presence of RNase sensitive components (including RNA-containing ICs) in their plasma (**Figures 1C, D**). As expected, patients with RNase-sensitive samples had higher serum levels of IFN-λ1, IFNα2a, and IP-10 (**Figures 4A–C**) than those samples without these components. Additionally, the presence of RNA components in plasma was associated with increased levels of circulating ICs (**Figure 4D**). Since complement consumption has been linked to the presence of ICs (17), we also assessed the levels of complement C3 in the RNase-insensitive and RNase-sensitive groups. As expected, the latter had reduced complement C3 levels (**Figure 4E**). Moreover, patients in the RNase-sensitive group had elevated anti-dsDNA levels (**Figure 4F**), indicating active disease status. Finally, RNase-sensitive plasma samples induced more neutrophil activation than the control samples as evidenced by the upregulation of CD11b and CD66b levels (**Figures 4G, H**).

## Discussion

Neutrophil activation plays an essential role in the pathogenesis of SLE. However, the factors and mechanisms driving neutrophil activation in SLE have not been extensively investigated. In this study, we demonstrated that plasma samples from SLE patients, containing ICs enriched with RNA components, triggered *de novo* neutrophil activation via a TLR8-dependent mechanism. Furthermore, the presence of RNA components correlated with elevated circulating IFN levels and enhanced inflammation that is mediated by ICs.

We observed that plasma levels of neutrophil activation markers, including calprotectin, MPO-DNA, and NE-DNA complexes, were significantly elevated in patients with SLE as compared with HCs, consistent with prior work (10, 18), indicating neutrophil hyperactivation and NET formation in SLE. Regarding the potential stimuli promoting neutrophil activation in SLE, a previous study has shown that TLR7/8 activation is necessary for NET formation induced by RNP-containing ICs in pediatric SLE (9). Our earlier work has similarly demonstrated that RNP-containing ICs promoted neutrophil activation in a TLR7/8-dependent manner (13), shifting neutrophils from phagocytosis of ICs toward NET formation. Of note, these prior studies only focused on select components, such as isolated IgG, in mediating neutrophil activation, rather than their importance of those components in a complex matrix, like plasma. In the current study, we found that SLE plasma induced significant neutrophil activation in a process substantially dependent on FcγR and RNA recognition by TLR8. The former has been well documented by us (4, 7, 13), as well as other groups (9, 19). It is noteworthy that FcγRIIA inhibition demonstrates the highest potency compared to other inhibitors. This aligns with immune complexes being the primary drivers of neutrophil activation. Of note, immune complexes can activate neutrophils through both TLR-dependent and TLR-independent (primarily FcγR-mediated) mechanisms, why we see more inhibition using FcγRIIA inhibitor as compared to TLR inhibitors. Further, TLR inhibitors would only block downstream TLR signaling, whereas the FcγRIIA inhibitor would block both FcγRIIA and all downstream signaling, including TLR. Bonegio et al. previously observed that lupus neutrophil activation can be induced by soluble RNA-containing ICs but not by soluble DNA-containing ICs (19). Instead, DNA-containing ICs need to be immobilized to activate human neutrophils effectively, consistent with our prior findings (13).

To identify the specific TLR responsible for RNA recognition in neutrophils, our data highlighted a unique role for TLR8, with minimal to no effect from TLR7. This is further supported by our finding that human neutrophils primarily expressed TLR8 on the mRNA level, with limited expression of TLR7. Our findings align with the studies by Berger et al. (20) and Makni-Maalej et al. (21), where they reported that human neutrophils expressed more TLR8 than TLR7. Further, Makni-Maalej et al. demonstrated that TLR8, but not TLR7, was involved in the priming of human neutrophil ROS production. In mice, Bonegio and colleagues showed that SLE-derived ICs activate neutrophils to release ROS and chemokines in an FcγRIIA-dependent but TLR7- and TLR9-independent manner (19). Although our data demonstrated that TLR7 agonists barely induced neutrophil activation, we cannot completely rule out the role of TLR7 in neutrophil processing of RNA-containing ICs. To further explore this, continued studies may be conducted to test the role of TLR7 antagonists on plasma-induced neutrophil activation. Additionally, TLR7 or TLR8 knock-out neutrophil cell lines, such as HL-60 cells, could be used to determine the specific ligand for RNA component in lupus-derived ICs. Although soluble DNA-containing ICs cannot drive neutrophil activation, these ICs can induce response in plasmacytoid dendritic cells and B cells (19). These findings, along with ours, suggest that SLE patients with different autoantibody profiles may benefit from targeted therapies aimed at specific immune cells or TLR pathways. Lastly, while neutrophil phagocytosis of ICs usually signals through endosomal TLRs, future studies on other RNA sensors, including cytoplasmic RIG-I or MDA5, are also important to further understand RNA recognition in neutrophils in SLE.

In summary, we have shown that IC-mediated neutrophil activation is dependent on RNA recognition by TLR8 in SLE. These findings suggest that SLE patients exhibiting heightened neutrophil activation, or the presence of RNA-containing ICs may benefit from targeted TLR8 inhibition or other RNA-elimination strategies, which will complement existing approaches, such as inhibiting NET formation, modulating protein arginine deiminase (PAD) activity, and regulating mitochondrial function (6), to block neutrophil activation.

## Data Availability

All data are either presented in the current manuscript or available upon reasonable request to the corresponding author. However, due to IRB regulations, patient-related data will only be shared deidentified upon establishing a data sharing agreement.
