# Supplementary material for: Immune complexes-mediated activation of neutrophils in systemic lupus erythematosus is dependent on RNA recognition by toll-like receptor 8
Source: Front Immunol. 2024 Dec 24;15:1515469. doi: 10.3389/fimmu.2024.1515469 (PMC11703909; doi:10.3389/fimmu.2024.1515469)
Supplement: Supplementary file 1 [file DataSheet1.docx]

**Supplementary material**

**Supplementary Table 1. Patient demographics in cohort 1.**

| **Cohort** | **SLE** | **HC** |
| --- | --- | --- |
| Number (#) | 151 | 31 |
| Age years, median (range) | 38 (19-78) | 35 (23-70) |
| Female (%) | 93 | 55 |
| Race – White (%) | 54 | 74 |
| Race – African American (%) | 10 | 0 |
| Race – Asian (%) | 23 | 13 |
| Race – Others (%) | 13 | 13 |
| SLEDAI, median (range) | 4 (0-26) | N/A |
| History of nephritis (%) | 53 | N/A |
| Steroids current (%) | 49 | N/A |
| HCQ current (%) | 93 | N/A |
| Anti-dsDNA (%) | 80 | N/A |
| Anti-SmRNP (%) | 52 | N/A |

**Supplementary Table 2. Primer sequences used for RT-qPCR.**

| **Gene** | **Forward (5' to 3')** | **Reverse (5' to 3')** |
| --- | --- | --- |
| 18S | GAGGGAGCCTGAGAAACGG | GTCGGGAGTGGGTAATTTGC |
| TLR7 | CGAACACCACGAACCTCACC | CCCAGTGGAATAGGTACACAGTT |
| TLR8 | GACTACAGGAAGTTCCCCAAAC | AGATTTTGCAGCCCTTGAAATGA |
| TLR9 | GAAGGGACCTCGAGTGTGAA | CTGGAGCTCACAGGGTAGGA |

**Supplementary Figure 1. Levels of biomarkers associated with neutrophil activation in SLE patients.**


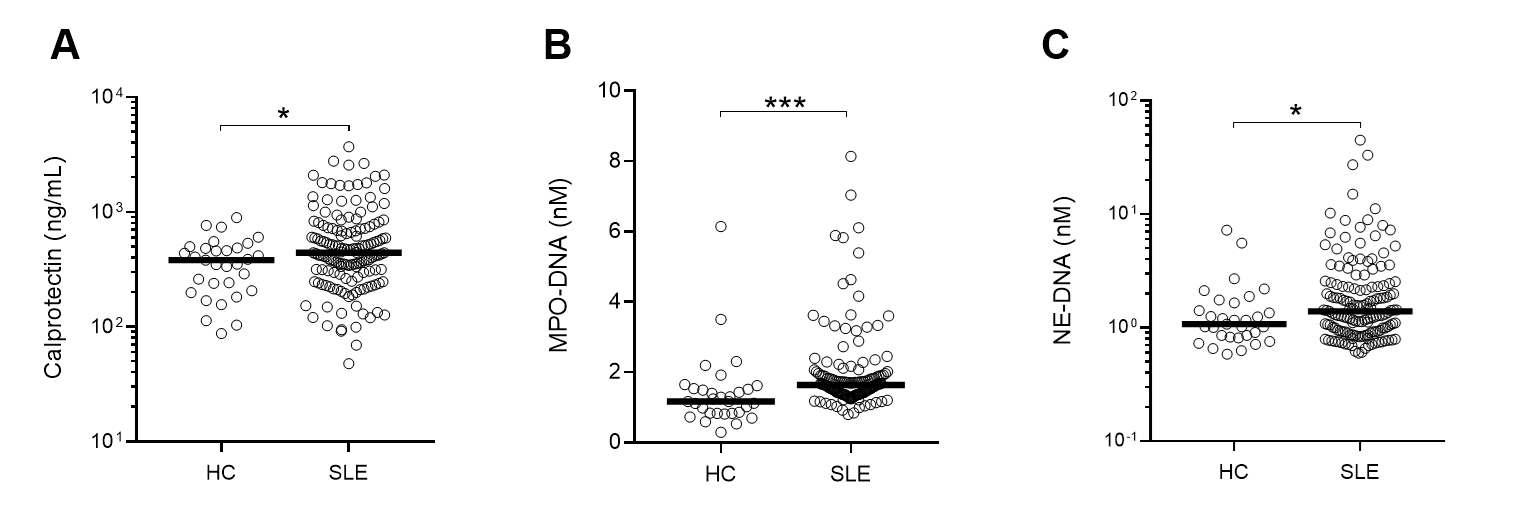


Plasma levels of calprotectin (A), MPO-DNA complexes (B), and NE-DNA complexes (C) were measured by ELISA in HCs (n=31) and patients with SLE (n=151). Statistical analyses were performed using the Mann-Whitney U-test; *p < 0.05, ***p < 0.001. Each circle represents an individual sample, with the bar representing the median of the group.

**Supplementary Figure 2. Primary neutrophil gating strategies.** Human neutrophils (Nph) were identified and gated using forward and side scatter properties, combined with the expression of CD66b.


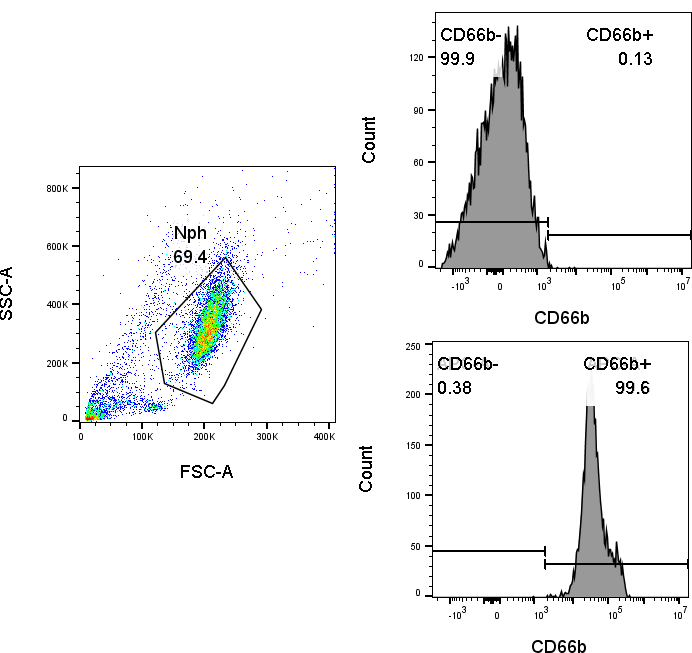


CD66b staining

No staining
